# Broad-spectrum antiviral activity of antisense oligonucleotides targeting GBF1 against SARS-CoV-2 and influenza viruses

**DOI:** 10.1016/j.isci.2026.114851

**Published:** 2026-01-29

**Authors:** Victoria Simanihuruk, Yurie Kida, Kosuke Takada, Harumi Yamaguma, Natsumi Kameoka, Itsuki Anzai, Shintaro Shichinohe, Satoshi Obika, Yuuya Kasahara, Tokiko Watanabe

**Affiliations:** 1 Department of Molecular Virology, Research Institute for Microbial Diseases, The University of Osaka, Osaka, Japan; 2 National Institutes of Biomedical Innovation, Health and Nutrition (NIBN), Osaka, Japan; 3 Center for Infectious Disease Education and Research (CiDER), The University of Osaka, Osaka, Japan; 4 Center for Advanced Modalities and DDS (CAMaD), The University of Osaka, Osaka, Japan; 5 Graduate School of Pharmaceutical Sciences, The University of Osaka, Osaka, Japan; 6 Institute for Open and Transdisciplinary Research Initiatives (OTRI), The University of Osaka, Osaka, Japan

**Keywords:** nucleic acids, virology, viral microbiology

## Abstract

Influenza viruses and severe acute respiratory syndrome coronavirus 2 (SARS-CoV-2) are respiratory pathogens that continue to challenge global health due to their efficient transmission and ability to circumvent virus-specific treatments. Targeting host factors that are essential for viral replication may enable the development of broad-spectrum antivirals with reduced resistance potential. Here, we used small interfering RNA (siRNA) to screen 91 host factors previously implicated in influenza virus replication and identified seven that were also required for SARS-CoV-2 replication. Of these, Golgi-specific brefeldin A-resistance factor 1 (GBF1), a guanine nucleotide exchange factor involved in coat protein complex I (COPI) vesicle trafficking, was also involved in human coronavirus 229E replication. We found that GBF1 relocated to sites of viral replication in SARS-CoV-2-infected cells. Using a computational design pipeline, we generated antisense oligonucleotides (ASOs) targeting GBF1. The lead candidate, GBF1-ASO#1502, potently inhibited influenza viruses and SARS-CoV-2 *in vitro*, with nanomolar half-maximal inhibitory concentration (IC_50_) values and favorable selectivity indices. GBF1-targeting ASOs thus represent a promising host-directed antiviral approach for controlling respiratory RNA viruses.

## Highlights

• GBF1 is a host factor for influenza, SARS-CoV-2, and HCoV-229E replication
• GBF1 knockdown suppresses SARS-CoV-2 viral growth and subgenomic mRNAs
• GBF1 relocates to viral replication sites during SARS-CoV-2 infection
• GBF1-targeting ASOs represent a potential host-directed antiviral strategy

## Graphical abstract

**Figure:**
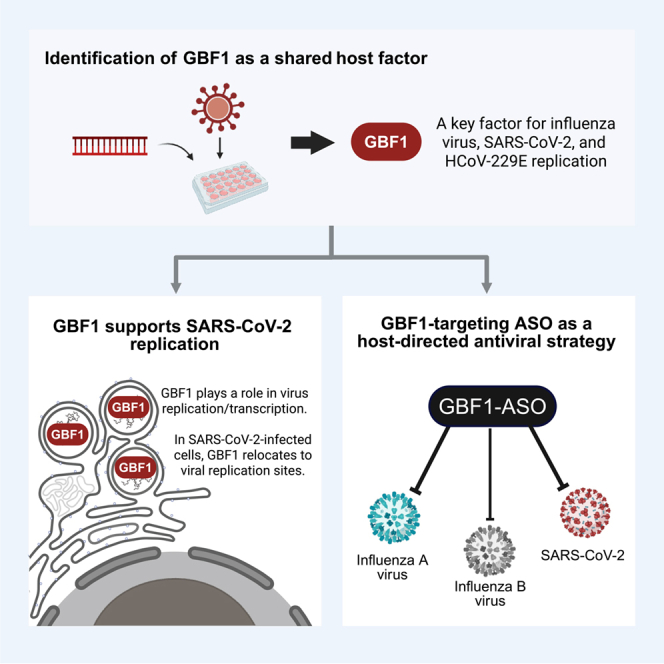

## Introduction

Respiratory RNA viruses such as influenza viruses and severe acute respiratory syndrome coronavirus 2 (SARS-CoV-2) have repeatedly caused widespread illness and death worldwide. Seasonal influenza alone is estimated to infect up to one billion people annually, with three to five million cases classified as severe and 290,000 to 650,000 deaths reported each year.^1^ Since its emergence in late 2019, SARS-CoV-2 has resulted in approximately 7.1 million deaths globally, as of November 19, 2025.^2^ Current countermeasures rely on virus-specific drugs and vaccines. Because these therapeutics target viral proteins, each pathogen requires a separate development pipeline, so for many newly emerging or re-emerging viruses, no effective antiviral treatments are available. Moreover, the emergence of drug-resistant variants diminishes the effectiveness of existing therapies.^3^^,^^4^^,^^5^ For instance, oseltamivir-resistant influenza A (H1N1) strains rapidly became dominant during the 2008–2009 season,^6^ and multiple monoclonal antibodies for SARS-CoV-2, including sotrovimab and casirivimab-imdevimab, have lost efficacy due to spike protein (S) mutations.^7^^,^^8^^,^^9^^,^^10^ These challenges underscore the need to develop antivirals that exhibit broad-spectrum activity and resilience to viral resistance.

Unlike bacteria, viruses are dependent on the host cellular machinery for their replication.^11^ Large-scale screening studies have identified numerous host proteins essential for the replication of diverse viruses.^12^^,^^13^^,^^14^^,^^15^^,^^16^^,^^17^ Systematic reviews and meta-analyses have revealed that some host factors are exploited by a range of viruses.^18^^,^^19^^,^^20^^,^^21^ Several small-molecule inhibitors targeting host proteins have demonstrated antiviral effects *in vitro* and *in vivo*.^22^^,^^23^^,^^24^^,^^25^^,^^26^^,^^27^^,^^28^ Host-directed approaches are increasingly being recognized as promising alternatives to virus-targeted therapies. By inhibiting a common host factor, it may be possible to develop a single therapeutic agent that is effective against multiple viruses, including newly emerging variants, and to minimize the risk of resistance associated with viral mutation.

Antisense oligonucleotides (ASOs) are synthetic, short nucleic acid molecules designed to hybridize with complementary messenger RNA (mRNA) sequences, leading to the degradation of the target transcript through RNase H activity. Chemical modifications can be introduced to improve stability, enhance binding specificity, and limit immune activation. Notably, ASOs can sometimes be delivered directly, without carrier systems, which can improve their tolerability and reduce immunogenicity compared to other RNA-targeting modalities.^29^^,^^30^ However, off-target effects and suboptimal knockdown efficiency were reported with early-generation ASOs. Therefore, new ASO design approaches have incorporated advanced computational frameworks, including mRNA secondary structure prediction, binding affinity calculations, and sequence-based toxicity risk assessment, often using *in silico* algorithms that integrate multiple evaluation parameters.^31^^,^^32^ For instance, gapmer-type ASOs consisting of 18 nucleotides, with bridged nucleic acids at both ends, have been designed using a 32-point evaluation scheme.^33^^,^^34^ This design strategy has been successfully applied in cancer research, targeting SRRM4 in lung cancer and SYT13 in gastric cancer.^33^^,^^34^ However, the application of such strategies to viral infectious diseases, particularly for targeting host factors, remains largely unexplored.

In the present study, we evaluated host factors previously implicated in influenza virus replication for their roles in SARS-CoV-2 replication. Ninety-one such factors^35^ were subjected to a small interfering RNA (siRNA)-based screening, revealing seven that were required for the replication of both influenza virus and SARS-CoV-2. Of these, GBF1 (Golgi-specific brefeldin A-resistance factor 1) was also involved in human coronavirus 229E (HCoV-229E) replication. GBF1 is a guanine nucleotide exchange factor (GEF) that plays an important role in the formation of coat protein complex I (COPI) vesicles responsible for retrograde transport^36^^,^^37^^,^^38^^,^^39^^,^^40^ through ADP-ribosylation factor (ARF) activation.^36^^,^^37^^,^^38^ By designing and evaluating ASOs targeting GBF1, we reveal its ability to suppress influenza A virus and SARS-CoV-2 replication *in vitro*. These results identify GBF1 as a pivotal host factor and support its potential as a therapeutic target for broad-spectrum antiviral strategies against multiple respiratory RNA viruses.

## Results

### Identification of host factors involved in SARS-CoV-2 and HCoV-229E replication via siRNA screening

Our previous study^35^ mapped viral-host interactions in cells infected with influenza virus. Eleven viral proteins, each fused with a FLAG tag, were expressed individually in human embryonic kidney (HEK) 293 cells and isolated using anti-FLAG immunoprecipitation. This yielded 1,292 candidate host proteins. A subsequent siRNA functional screen revealed 91 candidates whose depletion reduced viral titers by ≥3 log_10_ units without affecting cell viability.^35^

**Figure 1:**
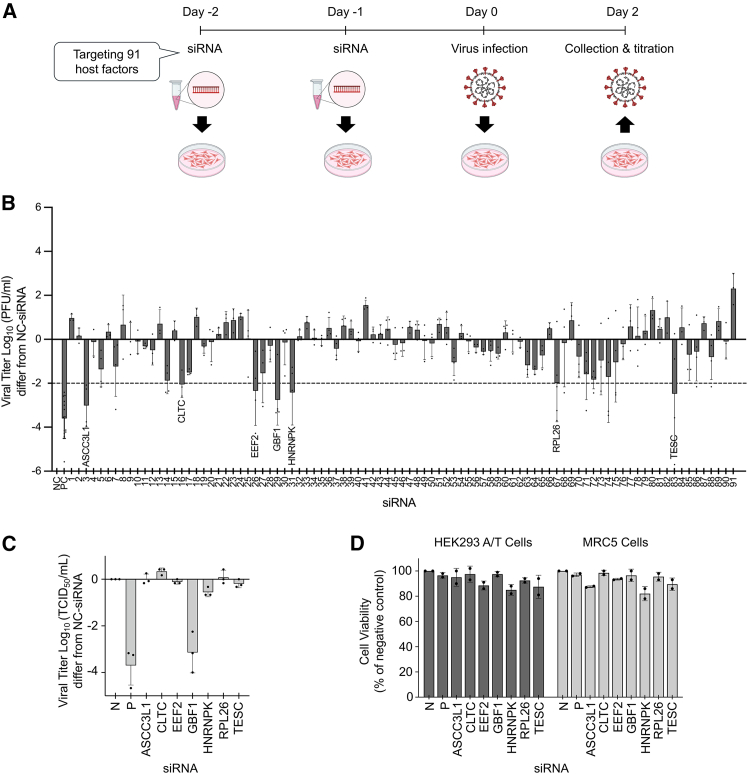
Identification of host factors involved in SARS-CoV-2 and HCoV-229E replication (A) Schematic overview of the siRNA screening. HEK293 A/T cells were seeded in 24-well plates and transfected twice with siRNAs targeting 91 host genes previously identified as being involved in influenza virus replication. Cells were then infected with SARS-CoV-2 (100 plaque-forming unit [PFU]/100 μL) one day after the second transfection. Supernatants were collected at 2 days post-infection (dpi) and titrated by plaque assay. (B) Results of the SARS-CoV-2 siRNA screen. The 91 host factors were divided into four batches, each including a non-targeting siRNA as a negative control (N) and siRNA targeting SARS-CoV-2 nsp12 as a positive control (P). Viral titers were calculated based on the difference between each siRNA and its corresponding negative control. Each dot represents the mean of duplicate wells from a single independent experiment. Data are presented as the mean ± standard deviation (SD) of at least three independent experiments. (C) The seven host factors identified in the SARS-CoV-2 siRNA screen were further examined in HCoV-229E, along with the negative control siRNA (N) and positive control siRNA (P) targeting the HCoV-229E N gene. Following the same method described in (A), MRC-5 cells were infected with HCoV-229E (50 tissue culture infectious dose (TCID_50_)/100 μL). Supernatants were collected at 3 dpi and titrated by TCID_50_ assay. Viral titers were calculated based on the difference between each siRNA and the negative control. Each dot represents the mean of duplicate wells from a single independent experiment. Data are presented as the mean ± SD of three independent experiments. (D) Cell viability was measured in duplicate wells across two independent experiments using CellTiter-Glo following siRNA transfection. See also Figures S1 and S2.

To determine whether these influenza virus-associated host factors also support the replication of other respiratory viruses, we performed an siRNA screen of these 91 host factors for their involvement in SARS-CoV-2 replication. The 91 siRNAs were introduced into HEK293 cells, which express human ACE2 and TMPRSS2 (HEK293 A/T), via both reverse and forward transfection. Two days after SARS-CoV-2 infection, culture supernatants were harvested for viral titer quantification using plaque assays (Figure 1A). The siRNAs were tested in four batches, each including a non-targeting control and an siRNA targeting non-structural protein 12 (nsp12) of SARS-CoV-2 as a positive control. Seven siRNAs targeting host genes (*ASCC3L1* [no. 3], *CLTC* [no. 16], *EEF2* [no. 26], *GBF1* [no. 29], *HNRNPK* [no. 31], *RPL26* [no. 67], and *TESC* [no. 83]) suppressed viral replication by at least 2 log_10_ units relative to the negative control (NC) (Figure 1B), while maintaining ≥85% cell viability (Figure 1D).

To test whether these genes are broadly required for coronavirus replication, we examined their roles in HCoV-229E replication in MRC-5 (Medical Research Council cell strain 5) human lung fibroblast cells. Of the seven genes, only GBF1 knockdown caused a substantial decline in virus replication (by 3.14 ± 0.88 log_10_ units) relative to the NC (Figure 1C), with 96.5% cell survival (Figure 1D).

These findings identify GBF1 as a conserved host factor involved in the replication of influenza virus, SARS-CoV-2, and HCoV-229E.

### GBF1 knockdown impairs SARS-CoV-2 replication

To assess the effect of GBF1 knockdown on SARS-CoV-2 replication, we confirmed the duration of GBF1 suppression following siRNA transfection. HEK293 A/T cells were transfected twice with either GBF1-targeting or control siRNAs. Cells were then harvested at various time points post-transfection for western blot analysis. GBF1 expression remained strongly suppressed for up to 72 h after the second siRNA transfection (Figure S1A), covering the time window of subsequent infection assays.

**Figure 2:**
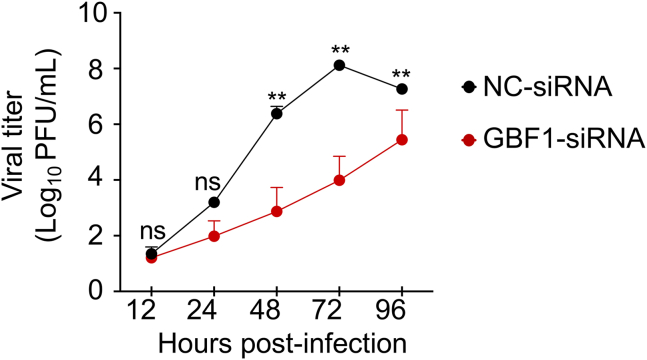
GBF1 knockdown impairs SARS-CoV-2 replication HEK293 A/T cells were transfected twice with either negative control siRNA (NC-siRNA) or GBF1-targeting siRNA (GBF1-siRNA). Viral growth kinetics were assessed after infection with SARS-CoV-2 at a low MOI of 0.0003. Supernatants were collected at the indicated time points, and viral titers were measured using plaque assays. Data are presented as the mean ± SD of triplicate wells from three independent experiments. Statistical analysis was performed using a two-way ANOVA followed by Bonferroni’s multiple comparisons test (ns: not significant; ∗*p* < 0.05; ∗∗*p* < 0.01).

When these cells were infected with SARS-CoV-2 at a low multiplicity of infection (MOI) (MOI ∼0.0003, as in the initial siRNA screen), viral titers in the supernatants were monitored at 12, 24, 48, 72, and 96 h post-infection (hpi). Across three independent experiments with triplicate samples, GBF1 knockdown resulted in a significant decrease in viral yield, reaching a 4.13 ± 0.86 log_10_ reduction at 72 hpi compared with the control siRNA (*p* < 0.01) (Figure 2). A similar experiment with HCoV-229E showed that GBF1 knockdown was sustained for up to 96 h after the second siRNA transfection of MRC5 cells (Figure S1B).

### GBF1 is involved in post-entry events in the SARS-CoV-2 replication cycle

**Figure 3:**
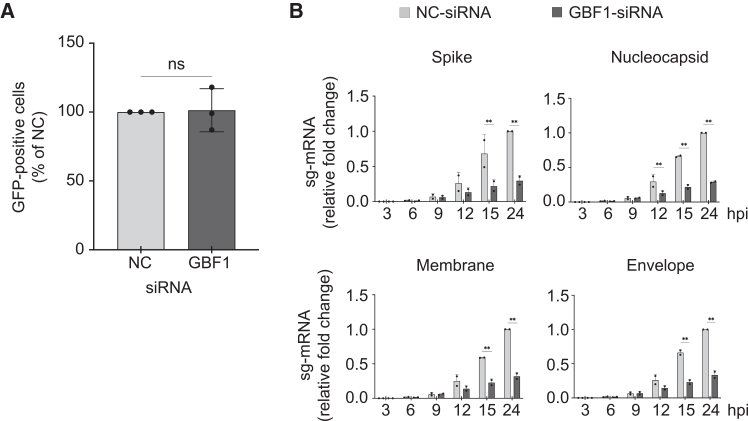
GBF1 is involved in post-entry events in the SARS-CoV-2 replication cycle (A) Pseudovirus entry assay. HEK293 A/T cells transfected with NC-siRNA or GBF1-siRNA were infected with VSV-SARS-CoV-2-S pseudovirus. GFP-positive cells were counted at 24 hpi and expressed as a percentage of NC-siRNA. Data are presented as the mean ± SD of duplicate wells from three independent experiments. Statistical significance was determined using a two-tailed unpaired Student’s *t* test (ns: not significant). (B) Viral subgenomic mRNA kinetics. HEK293 A/T cells transfected with NC-siRNA or GBF1-siRNA were infected with SARS-CoV-2 at an MOI of 10. The time of virus addition was defined as time 0. After a 1 h incubation to allow viral entry, the inoculum was removed and replaced with fresh medium. Cells were harvested at the indicated time points post-infection, and viral subgenomic-mRNAs (sg-mRNAs) encoding the spike (S), nucleocapsid (N), membrane (M), and envelope (E) proteins were quantified by RT-qPCR. Data are presented as fold change relative to NC-siRNA at each time point and represent the mean ± SD of triplicate wells from two independent experiments. Statistical analysis was performed using a two-way ANOVA followed by Šídák’s multiple comparisons test (ns: not significant; ∗*p* < 0.05; ∗∗*p* < 0.01).

To assess which stage of the viral replication cycle involves GBF1, we first examined whether GBF1 plays a role in SARS-CoV-2 entry using a vesicular stomatitis virus (VSV)-based pseudovirus system. VSV particles lacking the G gene (VSVΔG) and bearing the S of SARS-CoV-2 (VSV-SARS-CoV-2-S) were used to infect HEK293 A/T cells transfected with GBF1-siRNA or control siRNAs. The number of GFP-positive cells at 24 hpi was quantified to determine the infectivity of VSV-SARS-CoV-2-S. We found that the number of GFP-positive cells did not differ significantly between groups, indicating that GBF1 does not play a role in virus entry (Figure 3A).

We next assessed whether GBF1 influences SARS-CoV-2 replication and transcription by measuring the temporal dynamics of subgenomic mRNA (sg-mRNA) as a product of the replication/transcription process. GBF1-depleted cells were infected with SARS-CoV-2 at a high MOI (MOI = 10), and total RNA was extracted at 0, 3, 6, 9, 12, 15, and 24 hpi. sg-mRNA levels for the S, nucleocapsid (N), membrane (M), and envelope (E) genes were quantified by quantitative reverse-transcription PCR (RT-qPCR). GBF1 depletion reduced sg-mRNA levels for all four genes (70.6%, 71.1%, 68.1%, and 66.5% reduction relative to the NC at 24 hpi for S, N, M, and E, respectively; *p* < 0.01) (Figure 3B). In addition, western blot analysis of the same time points showed a general reduction in S, N, M, and E protein levels in GBF1-depleted cells, although the differences were not statistically significant (Figure S2A and S2B). These data indicate that GBF1 promotes SARS-CoV-2 replication/transcription, indirectly affecting viral protein synthesis.

### GBF1 re-localizes from the Golgi to viral replication sites upon SARS-CoV-2 infection

**Figure 4:**
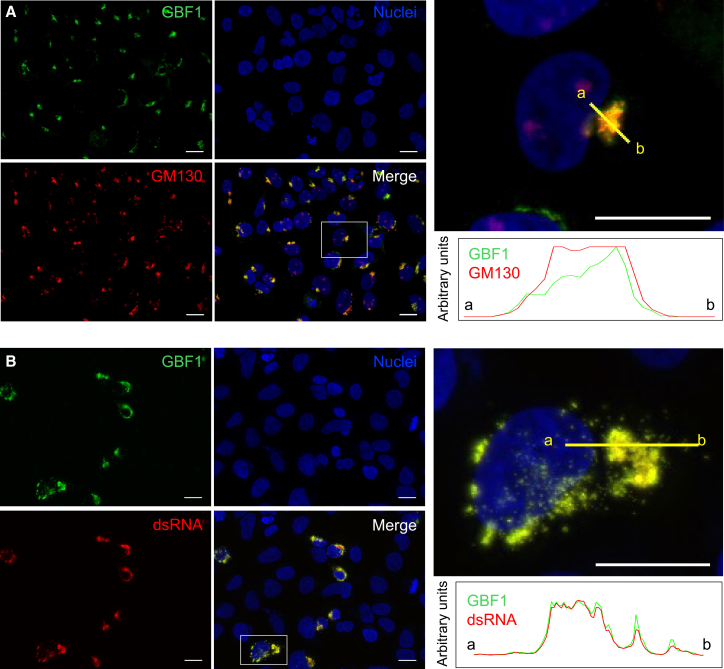
GBF1 is recruited from the Golgi to SARS-CoV-2 replication sites (A) Immunofluorescence of uninfected HEK293 A/T cells stained with antibodies against GBF1 (green) and GM130 (cis-Golgi marker, red), with Hoechst (blue) as a nuclear counterstain. (B) In HEK293 A/T cells infected with SARS-CoV-2 at an MOI of 2 and fixed at 6 hpi, GBF1 re-localized to cytoplasmic puncta that co-localized with double-stranded RNA (dsRNA, red), a marker of active viral replication. Insets show the boxed areas of each panel. The plots indicate the relative fluorescence intensity of individual channels corresponding to each line. Scale bars, 20 μm. See also Figure S3.

GBF1 is primarily localized at the *cis*-Golgi under normal conditions.^37^ To confirm its subcellular localization in uninfected cells, we performed immunofluorescence staining of HEK239 A/T cells. As expected, GBF1 co-localized with a *cis*-Golgi marker, GM130, displaying a characteristic perinuclear, dot-like distribution in the cytoplasm (Figure 4A).

Since GBF1 was found to be involved in SARS-CoV-2 replication and transcription, we next explored potential changes in its localization upon SARS-CoV-2 infection and whether it localized to the viral replication complex. Following SARS-CoV-2 infection at an MOI of 2, the HEK293 A/T cells were fixed with 4% paraformaldehyde (PFA) at 6 hpi and immunostained using an anti-GBF1 antibody (Figure 4B) or anti-GM130 antibody (Figure S3), anti-dsRNA as an active viral replication site marker, and counterstained with Hoechst to visualize nuclei. We observed that GBF1 co-localized with dsRNA, appearing as scattered puncta in the perinuclear region of the cytoplasm (Figure 4B). In contrast, GM130 showed a fragmented pattern and did not co-localize with dsRNA (Figure S3). These results indicate that GBF1 translocates from the Golgi to active viral replication sites during SARS-CoV-2 infection, supporting its functional involvement in the viral replication process.

### Design and screening of ASOs targeting *GBF1* mRNA

**Figure 5:**
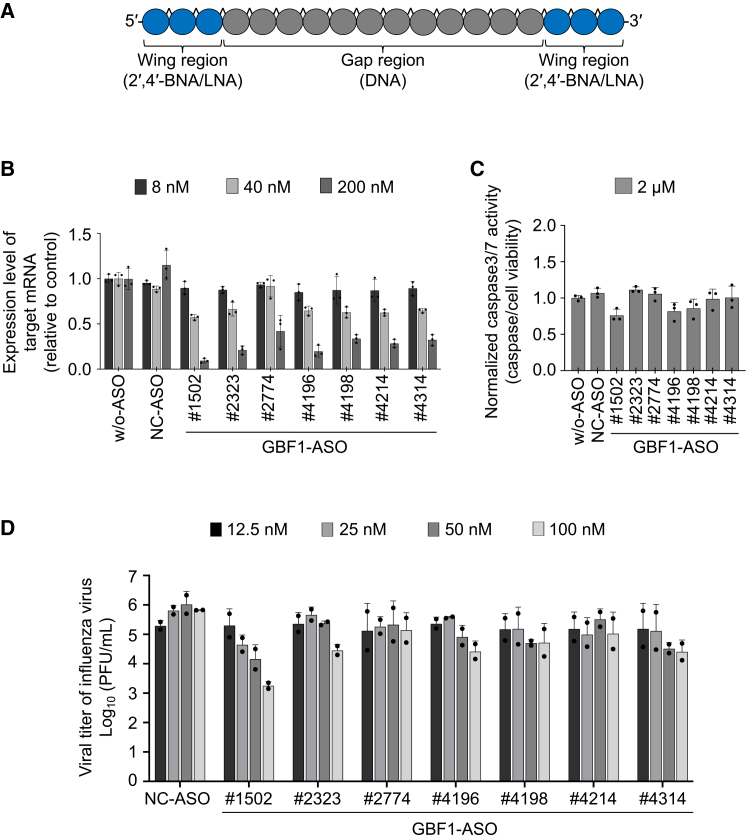
Screening of antisense oligonucleotides targeting GBF1 in HEK293 cells (A) Structure of ASOs targeting GBF1 mRNA (blue circle: 2′,4′-BNAs/LNAs; gray circle: DNA; ˆ: phosphorothioate linkage). (B) Evaluation of the concentration-dependent knockdown efficacy of the selected ASOs in HEK293 cells. The selected ASOs (8, 40, and 200 nM) were transfected into HEK293 cells using the CEM method, and the cells were incubated at 37°C with 5% CO_2_ for 24 h. Knockdown efficacy of GBF1 mRNA expression was calculated relative to the vehicle control upon ASO treatment. (C) The selected ASOs (2 μM) were transfected into HEK293 cells using the CEM method, and the cells were incubated at 37°C with 5% CO_2_ for 24 h. Caspase-3/7 activity was normalized to cell viability and expressed relative to the vehicle control (B and C). Data are presented as the mean ± SD from three independent experiments. (D) Screening of GBF1-ASOs against influenza A virus in HEK293 cells. HEK293 cells were reverse-transfected with various concentrations of GBF1-ASO using RNAiMAX reagent and then infected with influenza A virus (WSN strain). Supernatants were collected at 2 dpi, and viral titers were determined by plaque assays. Data are presented as the mean ± SD of duplicate wells from two independent experiments. w/o-ASO: without ASO (vehicle only), NC-ASO: negative control ASO. See also Figure S4.

To develop pharmacological agents that target GBF1, we designed a set of gapmer-type ASOs directed against human *GBF1* (*hGBF1*) mRNA, using a proprietary *in silico* algorithm optimized for RNase H-mediated transcript degradation. Each ASO was 18 nucleotides long, and both termini modified with 2′,4′-bridge nucleic acids (2′,4′-BNAs)/locked NAs (LNAs)^41^^,^^42^^,^^43^ to enhance binding affinity. All internucleotide phosphodiester (PO) bonds were substituted with phosphorothioate (PS) linkages to enhance nuclease resistance (Figure 5A). The design strategy excluded sequences predicted to have a high off-target risk.^31^ Subsequently, we selected ASOs that exhibited optimal binding affinity for RNA. ASOs that formed intramolecular stems or homodimers were excluded, resulting in a final library of 42 ASO candidates selected for synthesis and screening (Table S1).

To evaluate knockdown efficiency, we transfected the 42 ASO candidates targeting *hGBF1* mRNA into A549 cells (a human adenocarcinoma-derived cell line) using the Ca2+ enrichment of medium (CEM) method^44^ and used real-time PCR to measure the *hGBF1* mRNA expression levels at 24 h post-transfection (Figure S4A). Of the 42 candidates, 14 ASOs achieved ≥93% knockdown compared to the vehicle control. These 14 ASOs were then evaluated at various concentrations in A549 cells (Figure S4B), leading to the selection of seven ASO candidates with high knockdown efficiency and no cytotoxicity, as evaluated under the microscope, for further studies. To assess their effects in a different cell line and further evaluate cytotoxicity, these seven ASOs were tested in HEK293 cells. Knockdown efficiency was confirmed by real-time PCR, (Figure 5B) and cytotoxicity was assessed by measuring cell viability and caspase-3/7 activity (Figure 5C). All seven ASOs achieved dose-dependent knockdown with half-maximal inhibitory concentration (IC_50_) values below 100 nM and exhibited minimal cytotoxicity in HEK293 cells. Normalized caspase3/7 activity relative to cell viability indicated that GBF1-ASO#1502 exhibited the least cytotoxic effect, suggesting its potential as the safer option among the candidates (Figures 5B and 5C). From this initial screening, seven ASOs were selected as lead candidates based on both potency and safety profiles for evaluation of antiviral efficacy.

To evaluate their antiviral potential, the seven GBF1-ASOs were screened for effects on influenza A virus replication in HEK293 cells. Cells were transfected with each ASO at various concentrations (12.5–100 nM) and then infected with the virus. Among the tested ASOs, at 25 nM, only GBF1-ASO#1502 reduced viral titers by more than 90% (1.16 ± 0.52 log_10_ units) compared to the NC ASO (NC-ASO), with no detectable cytotoxicity (Figure 5D). Therefore, GBF1-ASO#1502 was selected as the lead compound for further evaluation.

### GBF1-ASO#1502 inhibits the *in vitro* replication of influenza viruses and SARS-CoV-2

**Figure 6:**
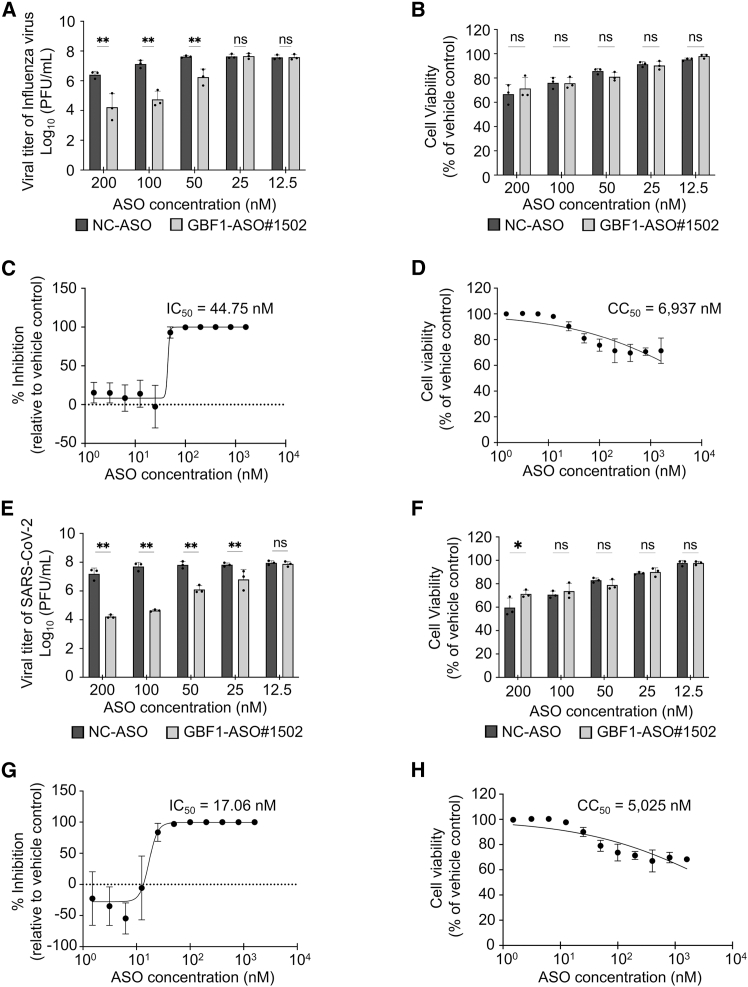
GBF1-ASO #1502 inhibits SARS-CoV-2 and influenza virus with high selectivity (A–D) Dose-dependent response of GBF1-ASO#1502 against influenza A virus in HEK293 cells. (A) Viral titer at 48 hpi measured by plaque assay. (B) HEK293 cell viability assessed using CellTiter-Glo. (C) Viral titer data from (A) are presented as the percent inhibition relative to the vehicle control, with a calculated IC_50_ = 44.75 nM and (D) CC_50_ = 6,937 nM (E–H) The same evaluations were performed in HEK239 A/T cells infected with SARS-CoV-2. (E) Viral titer at 72 hpi; (F) Cell viability; (G) Percent inhibition, with IC_50_ = 17.06 nM; and (H) CC_50_ = 5,025 nM. All data are presented as means ± SD from duplicate wells in three independent experiments. Statistical analyses for (A and B) and (E and F) were performed using a two-way ANOVA followed by Šídák’s multiple comparisons test (ns: not significant; ∗*p* < 0.05; ∗∗*p* < 0.01). See also Figures S5 and S6.

To quantify the antiviral efficacy and cytotoxicity of GBF1-ASO#1502, we transfected HEK293 or HEK293 A/T cells with various concentrations of ASO#1502 (0–1,600 nM), followed by infection with either influenza A virus or SARS-CoV-2. GBF1 protein knockdown in ASO-transfected cells at a concentration of 100 nM was confirmed in both cell lines (Figure S5). For influenza A virus, viral titers were measured by plaque assay at 48 hpi. A significant reduction in viral titer was observed starting at 50 nM, with a 1.38 ± 0.54 log_10_ decrease compared to cells treated with the NC ASO (Figure 6A). Cell viability remained above 80% at this concentration and did not differ significantly from that of cells treated with NC-ASO (Figure 6B). The IC_50_ of GBF1-ASO#1502 was 44.75 nM (Figure 6C), and the 50% cytotoxicity concentration (CC_50_) was 6,937 nM (Figure 6D), resulting in a selectivity index (SI = CC_50_/IC_50_) of >155.

Next, we assessed the antiviral efficacy of GBF1-ASO#1502 against SARS-CoV-2 under similar conditions. HEK293 A/T cells were transfected with GBF1-ASO#1502 at concentrations ranging from 0 to 1,600 nM, followed by SARS-CoV-2 infection. Viral titers were measured at 72 hpi. We observed a significant reduction in viral replication at 25 nM, with a 1.02 ± 0.69 log_10_ unit decrease compared to NC-ASO (Figure 6E). Cell viability at this concentration was 90%, indicating minimal cytotoxicity (Figure 6F). The calculated IC_50_ and CC_50_ values were 17.06 and 5,025 nM, respectively, yielding a SI (SI = CC_50_/IC_50_) of 294.54 (Figures 6G and 6H).

To evaluate its broad-spectrum antiviral potential, we tested GBF1-ASO#1502 against other influenza A (H3N2) and both influenza B lineages (B/Victoria and B/Yamagata). GBF1-ASO#1502 significantly reduced viral titers for all tested strains (>2 log_10_ for H3N2 and B/Victoria; approximately 1.9 log_10_ for B/Yamagata) at a concentration of 100 nM (Figure S6A). Combined with our data for influenza A (H1N1) and SARS-CoV-2 (Figure 6), these results demonstrate that GBF1-ASO#1502 has broad-spectrum activity across two viral families (*Orthomyxoviridae* and *Coronaviridae).*

To confirm that the antiviral effects are specifically due to GBF1 inhibition rather than off-target effects, we tested golgicide A (GCA), a well-characterized small molecule inhibitor of GBF1.^45^ GCA treatment reduced the replication of both influenza A virus and SARS-CoV-2 (Figures S6B and S6C), supporting GBF1 as an antiviral target through an independent inhibitory mechanism. However, GCA exhibited a narrow therapeutic window, with considerable cytotoxicity observed at concentrations required for antiviral activity, particularly for SARS-CoV-2 (Figures S6D and S6E). In contrast, GBF1-ASO#1502 demonstrated high selectivity indices (SI > 155 for influenza A virus; SI = 294.54 for SARS-CoV-2) with minimal cytotoxicity at effective concentrations (Figure 6). These findings highlight a key safety advantage of ASO-mediated GBF1 depletion over small molecule inhibition.

Together, our findings indicate that GBF1-ASO#1502 has broad-spectrum antiviral activity against influenza viruses and SARS-CoV-2 *in vitro*, with potent activity and favorable safety margins, supporting its potential as a host-directed antiviral agent.

## Discussion

Here, we performed an siRNA-based screen of 91 host factors previously identified as being involved in influenza virus replication^35^ and identified GBF1 as a shared host factor for influenza virus, SARS-CoV-2, and HCoV-229E replication. Employing SARS-CoV-2 as a model, we demonstrated that GBF1 plays an important role in the viral replication and transcription step. GBF1 knockdown impaired viral growth kinetics and reduced the expression of viral sg-mRNAs. The importance of GBF1 was further supported by immunofluorescence analysis showing its co-localization with double-stranded RNA (dsRNA), a marker of active viral replication, and its relocalization from the Golgi to viral replication sites in virus-infected cells (Figure 4)**.** To translate these findings into a therapeutic strategy, we designed ASOs targeting GBF1 using a computational algorithm optimized for potency and specificity. Among the candidates, GBF1-ASO#1502 demonstrated broad-spectrum activity across two viral families (*Orthomyxoviridae* and *Coronaviridae*) at nanomolar values and a favorable safety profile compared to GCA (Figures 6 and S6). Taken together, these results support the concept that GBF1 is a critical and druggable host factor and highlight the potential of host-targeted ASO therapeutics against multiple respiratory RNA viruses.

As a GEF, GBF1 facilitates ARF activation.^36^^,^^37^^,^^38^ ARF, a small guanosine triphosphatase (GTPase), plays a key role in regulating retrograde vesicle transport, which is crucial for COPI-coated vesicle formation during transport between the Golgi apparatus and the endoplasmic reticulum (ER).^36^^,^^37^^,^^38^^,^^39^^,^^40^ GBF1 primarily functions at the cis-Golgi and ER-Golgi intermediate compartment (ERGIC), where it plays a pivotal role in maintaining Golgi architecture.^37^^,^^45^^,^^46^^,^^47^ Previous studies have demonstrated that GBF1 is involved in the replication of several positive-stranded RNA viruses, including enteroviruses, flaviviruses, and coronaviruses.^18^^,^^48^^,^^49^^,^^50^^,^^51^ In the case of coronaviruses, such as mouse hepatitis coronavirus (MHV) and SARS-CoV, brefeldin A (BFA) treatment—a broad ARF-GEF inhibitor that affects GBF1, BIG1, and BIG2^52^—reduces viral RNA synthesis due to decreased numbers of double-membrane vesicles (DMVs) in virus-infected cells, suggesting that GBF1 contributes to membrane remodeling and the formation of replication organelles.^53^^,^^54^ Consistent with this, knockdown of ARFs or subunits of the COPI complex, both functionally linked to GBF1, has been shown to interfere with MHV, SARS-CoV, and HCoV-229E replication.^53^^,^^55^^,^^56^ Moreover, dominant-negative Arf1 overexpression strongly suppresses MHV replication, supporting a model in which GBF1 facilitates the maturation of viral replication organelles by mediating membrane lipid trafficking from the ERGIC to the ER.^53^ Although GBF1 inhibition has been reported to reduce SARS-CoV-2 replication,^57^ the precise mechanism has yet to be elucidated. Here, we demonstrated that suppression of GBF1 significantly reduces sg-mRNA synthesis in SARS-CoV-2-infected cells, implicating GBF1 in the replication stage. Furthermore, immunofluorescence analysis revealed the relocalization of GBF1 to viral replication sites, providing direct evidence of its functional involvement. While previous studies have shown that GBF1 does not co-localize with replication complexes in MHV-infected cells,^53^ our findings reveal that GBF1 is actively recruited to replication sites during SARS-CoV-2 replication, indicating that GBF1 may play virus-specific roles among coronaviruses. Our observations suggest that GBF1 promotes replication complex formation by coordinating membrane flow or scaffolding host and viral components. However, the precise molecular mechanism by which GBF1 facilitates viral replication remains to be fully elucidated. Future studies employing co-immunoprecipitation or proximity labeling approaches could help define whether GBF1 directly interacts with viral dsRNA to support viral RNA synthesis, facilitates DMV remodeling through direct interactions with nsps (such as nsp3 or nsp4) that are components of the replication complex,^58^ or both. In addition, GBF1 overexpression studies, including dominant-negative GBF1 overexpression, could provide further mechanistic insights into its role in the viral life cycle.

ASOs are chemically synthesized short nucleic acids that specifically bind to complementary RNA sequences via base pairing and regulate gene expression through RNase H-mediated degradation or steric hindrance. Their advantages include enhanced stability, specificity, and an extended *in vivo* half-life through chemical modifications such as LNA. In addition, unlike other RNA targeting approaches, ASOs can be administered without the need for delivery vehicles.^29^^,^^59^ These features simplify administration and reduce the risk of inflammation, making ASOs an attractive option for antiviral therapy. Research on ASOs targeting viral genomes has progressed rapidly in recent years. For SARS-CoV-2, ASOs targeting the 5′ untranslated region (5′-UTR) or the main protease (Mpro) coding region have shown efficacy in cell-based and animal models.^60^^,^^61^^,^^62^ Similarly, ASOs targeting the influenza virus genome have shown potent antiviral activity.^63^ These studies highlight the high specificity and therapeutic potential of genome-targeting ASOs, but they also underscore a key limitation: each ASO must be custom-designed and produced for individual viral pathogens. In contrast, our study aimed to develop ASOs targeting host factors that are shared among multiple viruses. We focused on GBF1, a key host protein for several RNA viruses, and designed 42 gapmer-type ASOs targeting its transcript. Among them, GBF1-ASO#1502 exhibited nanomolar IC_50_ values with minimal cytotoxicity against influenza virus and SARS-CoV-2 (Figure 6). To our knowledge, this is one of the first studies to demonstrate that ASOs targeting a host factor can exert cross-family antiviral effects against distinct RNA viruses, highlighting the feasibility of a host-directed, virus-independent approach. Our findings underscore the therapeutic potential of host-targeted ASOs as a broad-spectrum antiviral platform. Unlike virus-specific ASOs, host-directed ASOs can be applied across virus families, providing a flexible countermeasure against emerging and re-emerging viral infectious diseases. While their antiviral potency may be moderate compared to virus-specific agents, they could serve as early interventions to slow the spread of infection and act as a bridge until virus-specific vaccines or therapies become available. Future studies should focus on optimizing *in vivo* dosing and delivery and ensuring safety and effectiveness in animal models. Addressing these challenges will pave the way for host-targeted ASO therapeutics as powerful strategy for global infectious disease preparedness.

### Limitations of the study

While our study validates GBF1 as a key host factor for viral replication *in vitro*, several important next steps remain. Although host-directed therapies generally present a higher barrier to viral resistance than direct-acting antivirals, serial virus passaging experiments in the presence of sub-inhibitory concentrations of GBF1-ASO will be important to assess the potential for resistance evolution and to confirm long-term therapeutic durability.

Comprehensive *in vivo* validation will be essential for clinical translation. Future studies should evaluate safety, therapeutic efficacy, pharmacokinetics, and pharmacodynamics in animal models. Validation in primary human airway epithelial cultures or organoids would provide important preclinical data in physiologically relevant systems. In addition, antiviral potency against a broader range of viruses should be evaluated to confirm the potential of GBF1-ASO as a broad-spectrum, host-directed antiviral therapy.

We acknowledge the challenge of translating host-targeted approaches from *in vitro* to *in vivo*, as many fail due to pharmacokinetic, delivery, or compensatory adaptation issues. We designed our LNA/gapmer ASOs to address these limitations through enhanced stability, specificity, extended half-life, and independence from delivery vehicles. Nevertheless, rigorous *in vivo* studies will be essential to validate their therapeutic potential.

## Resource availability

### Lead contact

Further information and requests for resources and reagents should be directed to and will be fulfilled by the lead contact, Tokiko Watanabe (email: tokikow@biken.osaka-u.ac.jp).

### Materials availability

This study did not generate any new unique reagents. The antisense oligonucleotides (ASOs) targeting GBF1 described in this work are available upon reasonable request and completion of a Material Transfer Agreement (MTA).

### Data and code availability

All data reported in this paper will be shared by the lead contact upon request. This paper does not report original code. Any additional information required to reanalyze the data reported in this paper is available from the lead contact upon request. Raw western blot images are available in supplemental information, Data S1.

## Acknowledgments

We thank Susan Watson for scientific editing. We thank Mikiko Tanaka for excellent technical support and Kanako Hiromatsu for secretarial assistance. We thank the National Institute of Infectious Diseases (NIID) for providing SARS-CoV-2 (Wuhan strain hCoV-19/Japan/TY-WK-521/2020) and influenza viruses (A/Victoria/361/2011[A/H3N2] and B/Massachusetts/02/2012 [B/Yamagata]) and Yoshihiro Kawaoka for the influenza A virus H1N1 WSN strain and influenza B strain virus B/Tokyo/UT-BB078/2017 (B/Victoria). We also thank Chikako Ono and Yoshiharu Matsuura for kindly providing the HEK293 A/T cell line and the SARS-CoV-2 plasmid expression vector and Nicholas Yamahoki for establishing the VSV-based SARS-CoV-2 spike pseudovirus system. This study was supported by a 10.13039/501100001691JSPS 10.13039/501100001691KAKENHI Grant-in-Aid for Scientific Research (B) (JP22H02521 [to T.W.]), Grant-in-Aid for Scientific Research (C) (JP24K09264 [to S.S.]), Grants-in-Aid for Early-Career Scientists (JP25K18814 [to K.T.]); JP21K15042 and JP24K18069 [to I.A.]), 10.13039/100009619AMED Research Program on Emerging and Re-emerging Infectious Diseases (JP19fk0108113 [to T.W.]), AMED under grant no. JP223fa627002 (to T.W.), AMED Advanced Research and Development Programs for Medical Innovation (AMED-CREST) (JP22gm1610010 [to T.W.]), the AMED Strategic Center of Biomedical Advanced Vaccine Research and Development for Preparedness and Response (AMED-SCARDA) (JP223fa727001 [to Y. Kasahara]), the 10.13039/100007449Takeda Science Foundation (to T.W.), and RIKAKEN HOLDINGS CO. Young Researcher Support Grant-in-aid (to K.T.). The study was also supported by the CiDER Cross-Departmental “Infectious Diseases” Research Promotion Program, The University of Osaka. Icons in Figure 1A and the graphical abstract figure were created with BioRender.com.

## Author contributions

T.W. directed the research. H.Y., N.K., S.O., and Y. Kasahara designed and performed the ASO preliminary screening (*in silico* and *in vitro*). Y. Kida and K.T. performed the ASO screening in virus-infected cells. V.S. performed the siRNA screening, all virological experiments, molecular analyses, IFA, and ASO evaluation in influenza- and SARS-CoV-2-infected cells. V.S., Y. Kida, K.T., I.A., and T.W. analyzed and interpreted the data. V.S., Y. Kasahara, and T.W. wrote the manuscript. All authors reviewed, commented on, and edited the final manuscript.

## Declaration of interests

The authors declare no competing interests.

## STAR★Methods

### Key resources table

**Table:** 

REAGENT or RESOURCE	SOURCE	IDENTIFIER
**Antibodies**		

Mouse monoclonal anti-Spike	Bio Matrix Research	CSW1-1805
Rabbit monoclonal anti-Nucleocapsid (Clone HL344)	Cell Signaling Technology	Cat#26369; RRID:AB_2927757
Mouse monoclonal anti-Membrane (Clone E5A8A)	Cell Signaling Technology	Cat#15333
Rabbit monoclonal anti-Envelope (Clone HL1443)	Genetex	Cat#GTX636915
Rabbit polyclonal anti-GBF1	Abcam	Cat#ab86071; RRID:AB_1925022
Mouse monoclonal anti-Beta actin (Clone 6D1)	Wako	Cat#010-27841; RRID:AB_2858279
Mouse monoclonal anti-GBF1	BD Biosciences	Cat#612116; RRID:AB_399487
Rabbit monoclonal anti-GM130	ABclonal	Cat#A11408; RRID:AB_2861561
Mouse monoclonal anti-dsRNA (Clone J2)	Thermo Fisher Scientific	Cat#10010200; RRID:AB_2651015
Secondary antibodies (Alexa Fluor series)	Thermo Fisher Scientific	Cat#A-21121; RRID:AB_2535764, A-11012; RRID:AB_2534079, A-21135; RRID:AB_2535774

**Bacterial and virus strains**		

SARS-CoV-2 (Wuhan strain hCoV-19/Japan/TY-WK-521/2020)	NIID	N/A
HCoV-229E	ATCC	VR-740
Influenza A virus A/WSN/33 (H1N1; WSN)	Prof. Yoshihiro Kawaoka (The University of Tokyo)	N/A
A/Victoria/361/2011(A/H3N2)	NIID	N/A
B/Tokyo/UT-BB078/2017 (B/Victoria)	Prof. Yoshihiro Kawaoka (The University of Tokyo)	N/A
B/Massachusetts/02/2012 (B/Yamagata)	NIID	N/A
VSVΔG-GFP pseudovirus	Kerafast	Cat#EH1019-PM

**Chemicals, peptides, and recombinant proteins**		

Lipofectamine (RNAiMAX transfection reagent)	Thermo Fisher Scientific	Cat#13778-15D
Ca^2+^ enrichment of medium (CEM) reagents	Hori et al., 2015^43^	N/A
2′,4′-BNA/LNA phosphorothioate-modified oligonucleotides	N/A	custom synthesis
Hoechst 33342 trihydrochloride, trigydrate	Thermo Fisher Scientific	Cat#H3570
Golgicide A	Selleck	Cat#S7266

**Critical commercial assays**		

CellTiter-Fluor™ cell viability assay kit	Promega	Cat#G6080
Caspase-Glo® 3/7 assay system	Promega	Cat#G8091
RNeasy Mini Kit	Qiagen	Cat#74106
PrimeScript™ FAST RT Reagent Kit with gDNA eraser	Takara Bio	Cat#RR092S/RR092A
SuperPrep® Cell Lysis & RT Kit for qPCR	TOYOBO	Cat#SCQ-101
PowerTrack™ SYBR™ Green Master Mix	Thermo Fisher Scientific	Cat#A46109
SeaPlaque	Lonza	Cat#50100
SeaKem	Lonza	Cat#50074

**Experimental models: Cell lines**		

HEK293 cells	JCRB	JCRB9068
HEK293 A/T (ACE2/TMPRSS2 stable line)	Prof. Yoshiharu Matsuura (The University of Osaka)	N/A
A549 cells	JCRB	JCRB0076
MRC-5 cells	ATCC	CCL-171
VEROE6/TMPRSS2	JCRB	JCRB1819
MDCK	Prof. Yoshihiro Kawaoka (The University of Tokyo)	N/A
LentiX-293T	Clontech	Cat#632180

**Oligonucleotides**		

Silencer Select negative control No.1 siRNA (NC-siRNA)	Thermo Fisher Scientific	Cat#43908433
Silencer Select pre-designed siRNAs targeting host factors No.1–91	Watanabe et al.,2014^34^	N/A
Silencer Select siRNA targeting the nsp12 gene of SARS-CoV-2	Chang et al., 2022^64^	N/A
Silencer Select siRNA targeting the N gene of HCoV-229E (Sense, 5′-GCUUAUAGGCUAUUGGAAU-3′ and Antisense, 5′- AUUCCAAUAGCCUAUAAGC-3′)	custom synthesized	N/A
Primer for SARS-CoV-2-5-leader (Forward, 5′-ACCAACCAACTTTCGATCTCTTGT-3′)	custom synthesized	N/A
Primer for sgRNA of SARS-CoV-2 spike (Reverse, 5′-GTCAGGGTAATAAACACCACGTG-3′)	custom synthesized	N/A
Primer for sgRNA of SARS-CoV-2 nucleocapsid (Reverse, 5′-TGCGTTCTCCATTCTGGTTACTG-3′)	custom synthesized	N/A
Primer for sgRNA of SARS-CoV-2 membrane (Reverse, 5′-TAGTACCGTTGGAATCTGCC-3′)	custom synthesized	N/A
Primer for sgRNA of SARS-CoV-2 envelope (Reverse, 5′-ATATTGCAGCAGTACGCACACA-3′)	custom synthesized	N/A

**Software and algorithms**		

Proprietary in silico ASO design algorithm	N/A	N/A
GraphPad Prism (version 9.4.0)	GraphPad Software	RRID:SCR_002798
ImageJ/Fiji	NIH	RRID:SCR_003070

### Experimental model and study participant details

#### Cell lines and culture conditions

HEK293 cells, HEK293 cells stably expressing human ACE2 and TMPRSS2 (HEK293 A/T), A549 cells, VEROE6/TMPRSS2 cells, and LentiX-293T cells were maintained in DMEM, while human lung fibroblast MRC5 cells and MDCK wells were maintain in EMEM and MEM10x, respectively. All cell cultures were maintained in their respective medium, supplemented with 10% FBS (or NCS for the MDCK cell line) and antibiotics under standard culture conditions (37°C, 5% CO_2_). The SARS-CoV-2, HCoV-229E, and influenza virus strains used in this study are listed in the key resources table. All experiments involving live viruses were performed in BSL-3 or BSL-2 containment facilities as appropriate.

### Method details

#### siRNA screening for host factors

Ninety-one host factors previously identified as influenza virus interactors^35^ were subjected to siRNA screening in HEK293 A/T cells infected with SARS-CoV-2. Cells were reverse- and forward-transfected with individual siRNAs (10 pmol/well), and then infected with SARS-CoV-2 at 100 PFU/100 μL. Supernatants collected at 2 dpi were analyzed by plaque assay. Each batch included non-targeting siRNA (negative control) and siRNA targeting SARS-CoV-2 nsp12 (positive control). The selected seven host factors were further subjected to screening in MRC5 cells infected with HCoV-229E. Cells were transfected using the same method, but siRNA targeting the N gene of HCoV-229E served as a positive control. Cells were infected with HCoV-229E at 50 TCID_50_/100 μL and the supernatants collected at 3 dpi were analyzed by performing TCID_50_ assay.

#### Validation of GBF1 knockdown

HEK293 A/T or MRC5 cells were transfected twice with GBF1-targeting siRNA or negative control siRNA. Cells were lysed at the indicated time points for Western blot analysis of GBF1 protein levels.

#### Viral growth kinetics assay

NC-siRNA- or GBF1-siRNA-transfected HEK293 A/T cells were infected at a low MOI of 0.0003 (adjusted to the screening inoculum). Supernatants were collected at multiple time points and viral titers in the supernatants were measured by performing plaque assays.

#### Western blotting

Samples were lysed with Laemmli buffer containing 5% 2-Mercaptoethanol, heated for 10 min at 100°C, and then chilled prior to SDS-polyacrylamide gel electrophoresis (SDS-PAGE) (5%–20% precast gel), transferred to 0.45-μm PVDF membranes and incubated with 5% skim milk in PBS containing 0.1% Tween 20 (PBS-T) overnight at 4°C. The membranes were then stained with primary antibody for 1 h at room temperature. Blots were rinsed with PBS-T four times for 5 min and stained with secondary antibody for 1 h at room temperature. Protein bands were detected using either ImmunoStar Zeta or ImmunoStar LD detection reagent and visualized by using the ChemiDoCXRS+ Imaging System. Band signals were then quantified using ImageJ.

#### Pseudovirus entry assay

To assess viral entry, VSV-based pseudoviruses bearing SARS-CoV-2 S (VSV-SARS-CoV-2-S) protein were used. HEK293 A/T cells transfected with GBF1 or control siRNA were infected with the VSV-SARS-CoV-2-S at 1000 IU/well the day after the second transfection, and GFP-positive cells were quantified at 24 hpi by means of fluorescence microscopy.

#### Temporal dynamics of transcriptional/translational expression of viral genes

Cells depleted of GBF1 were infected with SARS-CoV-2 at an MOI of 10. Total RNA was extracted at multiple time points and subjected to RT-qPCR targeting S, N, M, and E sg-mRNAs. The relative mRNA expression was calculated by using the ΔΔCt method and the data are presented as log2-fold changes. Another set of cell lysates was collected for SARS-CoV-2 structural protein analysis and analyzed by western blotting. Data are shown in the linear scale.

#### Immunofluorescence microscopy

Cells were infected with SARS-CoV-2 at an MOI of 2 and were fixed with 4% paraformaldehyde, permeabilized using 0.05% Triton X-100 in PBS for 10 min and were blocked with Blocking One solution for 30 min. Cells were then stained with antibodies against GBF1, GM130, and dsRNA in 0.05% Tween 20/5% Blocking One/PBS overnight, at 4°C. After washes with 0.05% Tween 20/PBS, the cells were incubated with the appropriate secondary antibodies diluted in 5% Blocking One in PBS with 0.05% Tween 20 for 2 h at room temperature. Nuclei were stained with Hoechst for at least 15 min at room temperature. Images were acquired with a fluorescence microscope (BZ-x810, Keyence).

#### Design and screening of antisense oligonucleotides

Gapmer-type ASOs (18 nt) targeting hGBF1 mRNA were designed using an *in silico* algorithm optimized for RNase H-mediated degradation. Chemically modified with 2′,4′-BNA/LNA ends and phosphorothioate linkages, 42 candidates were synthesized. The off-target risk of ASOs was analyzed using package version GGGenome (Retrieva, Inc., Fukuoka, Japan) and Database for Drug Development with Genome and RNA sequences (D3G) (https://d3g.riken.jp/). The RNA binding affinity of the ASOs was predicted by the GC% and *T*_m_-value prediction model.^32^ All Designed ASOs were synthesized and purified by Ajinomoto Bio-Pharma Services Japan GeneDesign, Inc. (Osaka, Japan). Knockdown efficiency was evaluated in A549 cells and HEK293 cells by RT-PCR, and cytotoxicity was assessed by using cell viability and caspase-3/7 assays.

#### Antiviral efficacy assays

Cells were reverse-transfected with GBF1-ASO#1502 at various concentrations, followed by infection with influenza A virus (WSN) (50 PFU/well), A/Victoria/361/2011(A/H3N2) (50 PFU/well), B/Tokyo/UT-BB078/2017 (B/Victoria), B/Massachusetts/02/2012 (B/Yamagata) (1000 PFU/well) or SARS-CoV-2 (100 PFU/well). Supernatants were collected at 48 hpi for influenza virus and at 72 hpi for SARS-CoV-2. Viral titers were determined by plaque assay, and cell viability was measured to calculate IC_50_ values, CC_50_ values, and selectivity indices.

#### Small molecule inhibitor assays

WSN virus or SARS-CoV-2 at an MOI of 0.001 was used to infect HEK293 cells or HEK293 A/T cells. At 1 hpi, the cells were washed and incubated with medium containing the indicated concentration of GCA. DMSO (final concentration 0.1%, corresponding to the DMSO concentration in the highest GCA treatment) was used as a control. Culture supernatants were harvested at 48 hpi for influenza and 72 hpi for SARS-CoV-2. Viral titers were measured by use of plaque assays and cell viability was measured by using CellTiter-Glo.

#### Cell viability

Cell viability assessments were performed in 96-well flat bottom plates. For siRNA transfection, HEK293 A/T and MRC5 cells were reverse- and forward-transfected with selected siRNAs, and cell viability was measured the following day. For ASO transfection, HEK293 A/T and HEK239 cells were reverse-transfected with ASO targeting GBF1 and with an ASO negative control, and cell viability was assessed the following day. For GCA, the viability of uninfected HEK239 and HEK239 A/T cells treated with GCA or DMSO was assessed at time points corresponding to the infection experiment (2 and 3 days, respectively). Cell viability was determined using the CellTiter-Glo assay system according to the manufacturer’s instructions.

### Quantification and statistical analysis

Statistical analyses were performed using GraphPad Prism software. Comparisons between two groups were made using unpaired two-tailed Student’s t-tests (Figure 3A). For datasets containing one independent variable in multiple groups, a one-way ANOVA followed by Dunnet’s multiple comparisons test was performed (Figures S6B–S6E). For multiple group comparisons, a two-way ANOVA with Bonferroni’s (Figure 2), or Šidák’s (Figures 3B, 6A, 6B, 6E, 6F, S2B, and S6A), multiple comparisons test was applied. ∗*p* < 0.05 and ∗∗*p* < 0.01 were considered statistically significant.
