# Supplementary material for: Broad-spectrum antiviral activity of antisense oligonucleotides targeting GBF1 against SARS-CoV-2 and influenza viruses
Source: iScience. 2026 Jan 29;29(3):114851. doi: 10.1016/j.isci.2026.114851 (PMC12930041; doi:10.1016/j.isci.2026.114851)

iScience, Volume *29*

Supplemental information

Broad-spectrum antiviral activity of antisense oligonucleotides targeting GBF1 against SARS-CoV-2 and influenza viruses

Victoria Simanihuruk, Yurie Kida, Kosuke Takada, Harumi Yamaguma, Natsumi Kameoka, Itsuki Anzai, Shintaro Shichinohe, Satoshi Obika, Yuuya Kasahara, and Tokiko Watanabe

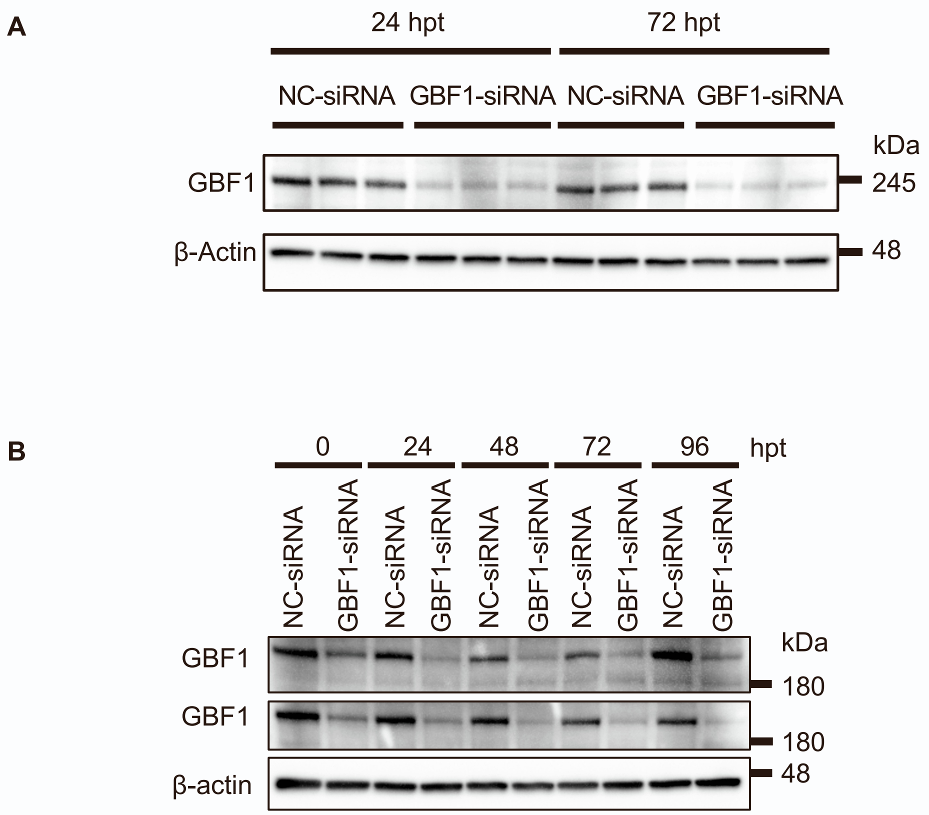

Figure S1. GBF1 knockdown confirmation. Related to Figure 1.
(A) HEK293A/T cells were transfected twice with either negative control siRNA (NC-siRNA) or GBF1-targeting siRNA (GBF1-siRNA). Cell lysates were collected at the designated time points post-transfection and analyzed by Western blotting to evaluate GBF1 expression, using β-actin as a loading control. Each siRNA was tested in triplicate wells. (h.p.t: hours post-second siRNA transfection). (B) MRC5 cells were transfected twice with either negative control siRNA (NC-siRNA) or GBF-1 siRNA (GBF1-siRNA). Cell lysates and supernatants were collected at the designated time points. Western blotting was performed using GBF1 or β-actin antibodies to confirm the knockdown effect in the cell lysates. Each siRNA was tested in duplicate wells. (h.p.t: hours post-second siRNA transfection).

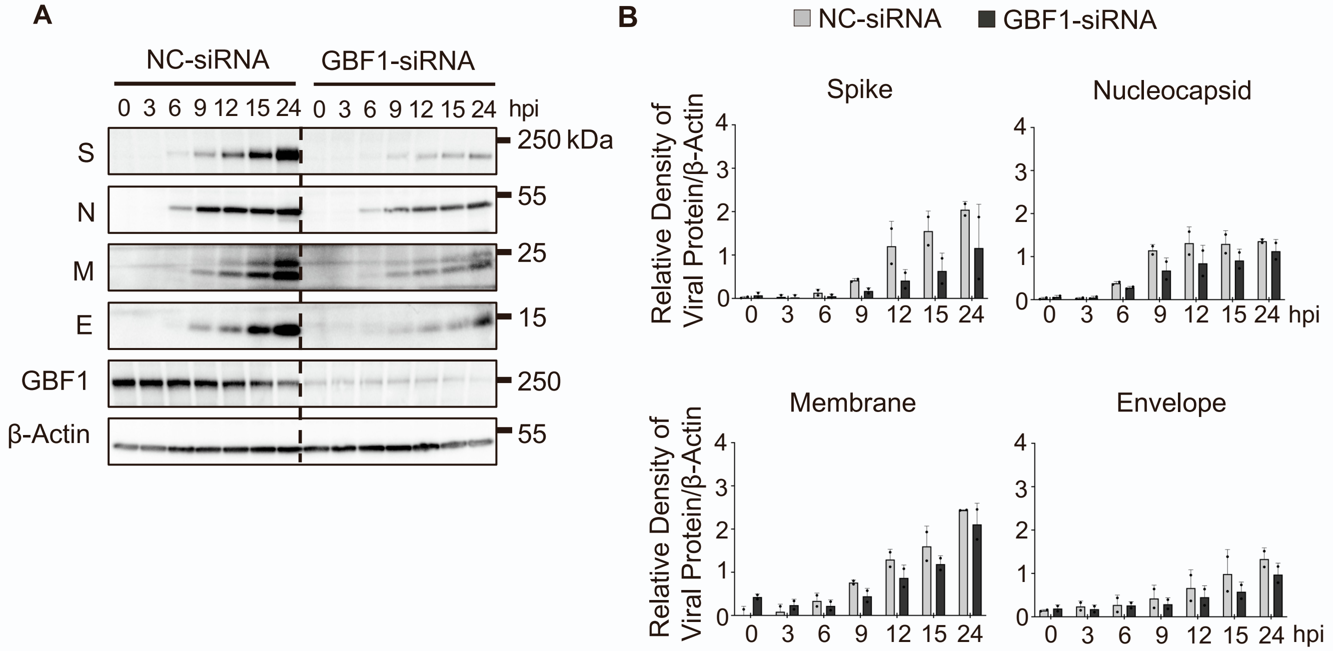

Figure S2. Temporal dynamics of SARS-CoV-2 structural proteins in GBF1-depleted cells. Related to Figure 3.

(A—B) Western blotting results of the four structural proteins (i.e., S, N, M, and E) in GBF1-depleted cells, followed by infection with SARS-CoV-2 at an MOI of 10. The time of virus addition was defined as time 0. Following a one-hour incubation for viral entry, the inoculum was removed and replaced with fresh medium. Cells were harvested at the indicated time points post-infection and subjected to western blotting analysis. (B) The relative protein levels were quantified by normalizing their values to β-actin. Each siRNA was tested in triplicate across two independent experiments. Statistical analysis was performed by using a two-way ANOVA followed by Šídák's multiple comparisons test (ns: not significant; $*p < 0.05$; $**p < 0.01$).

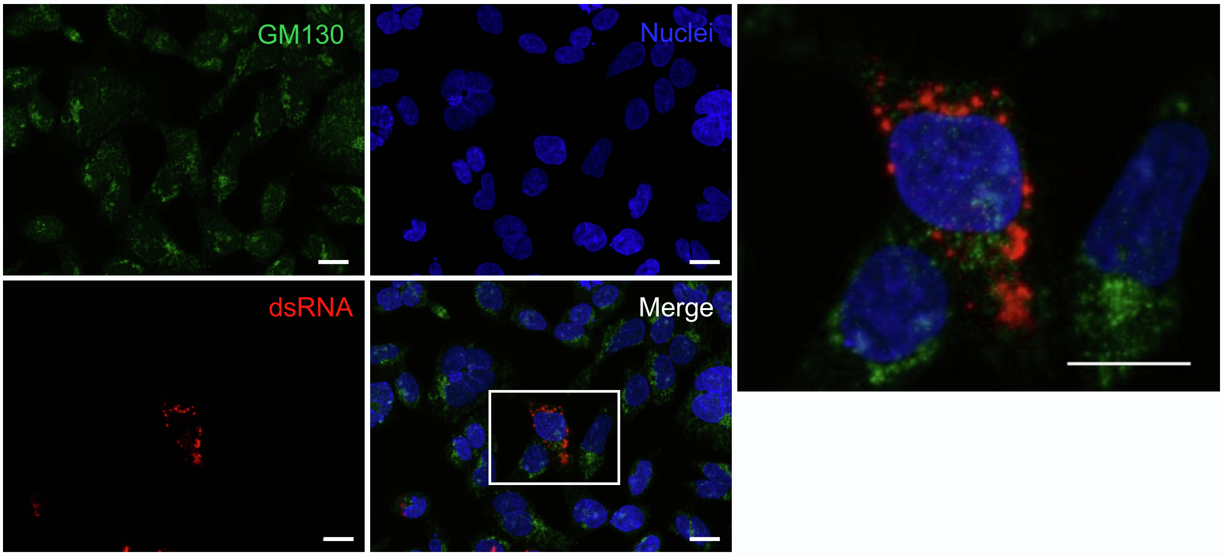

Figure S3. Golgi and dsRNA localization in SARS-CoV-2 infection. Related to Figure 4.
HEK239 A/T cells infected with SARS-CoV-2 at an MOI of 2 were fixed at 6 hpi and stained with antibody targeting GM130 (green), dsRNA (red), and Nuclei (blue). Bar = 20 μm.

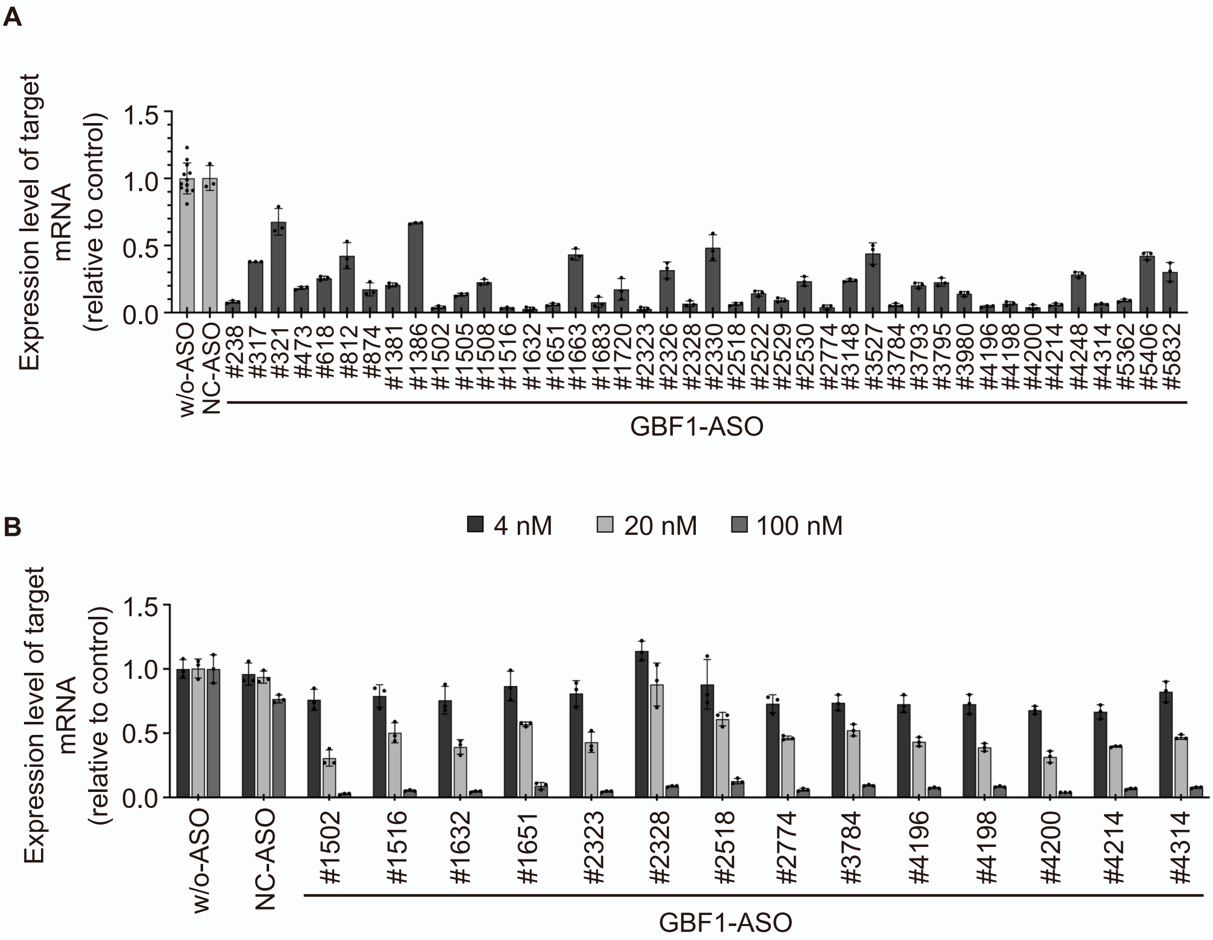

Figure S4. Screening of antisense oligonucleotides targeting GBF1 in A549 cells. Related to Figure 5.

(A) Primary screening of the designed ASOs according to the knockdown efficacy of GBF1 mRNA expression in A549 cells. The designed ASOs (100 nM) were transfected into A549 cells by using the CEM method and the cells were incubated at 37°C with 5% CO_2 for 24 h. Knockdown efficacy of GBF1 mRNA expression was calculated relative to the vehicle control upon ASO treatment. (B) Secondary screening of the selected ASOs according to the concentration-dependent knockdown efficacy of GBF1 mRNA expression in A549 cells. The selected ASOs (4, 20, and 100 nM) were transfected into A549 cells by using the CEM method and the cells were incubated at 37°C with 5% CO_2 for 24 h. Knockdown efficacy of GBF1 mRNA expression was calculated relative to the vehicle control upon ASO treatment. Data are means ± S.D. of three independent experiments.

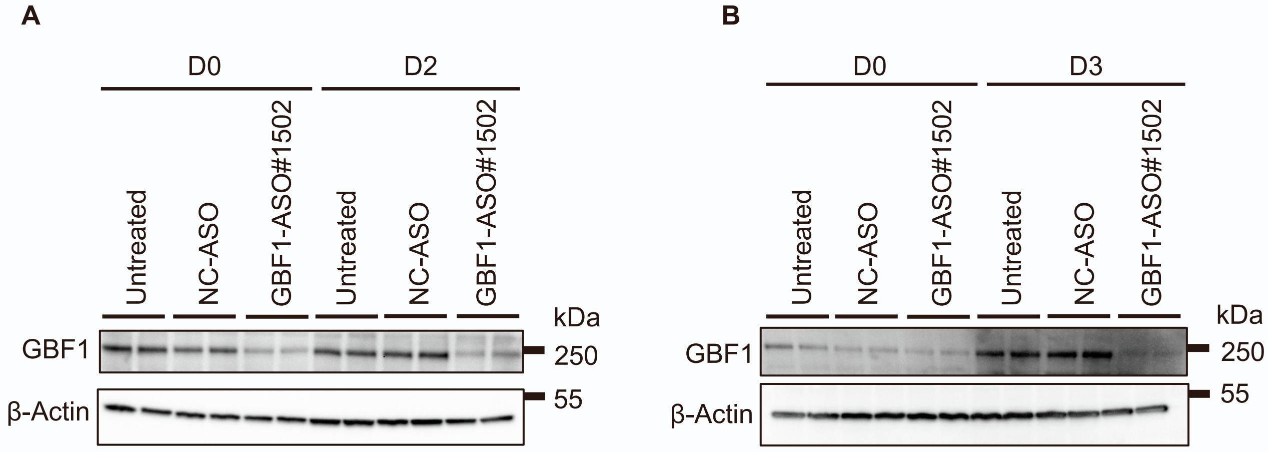

Figure S5. Knockdown confirmation of GBF1 ASO. Related to Figure 6.
(A) HEK293 cells and (B) HEK293 A/T cells were reverse-transfected with 100 nM negative control ASO (NC-ASO) or GBF1-ASO#1502. Cell lysates were collected at the indicated time points, without infection. Time points corresponded to the infection schedule: D0 = day of infection, D2 = 2 days post-infection, and D3 = 3 days post infection. Western blotting was performed using GBF1 or β-actin antibody to confirm the knockdown effect in each cell lysate. Each condition was tested in duplicate wells.

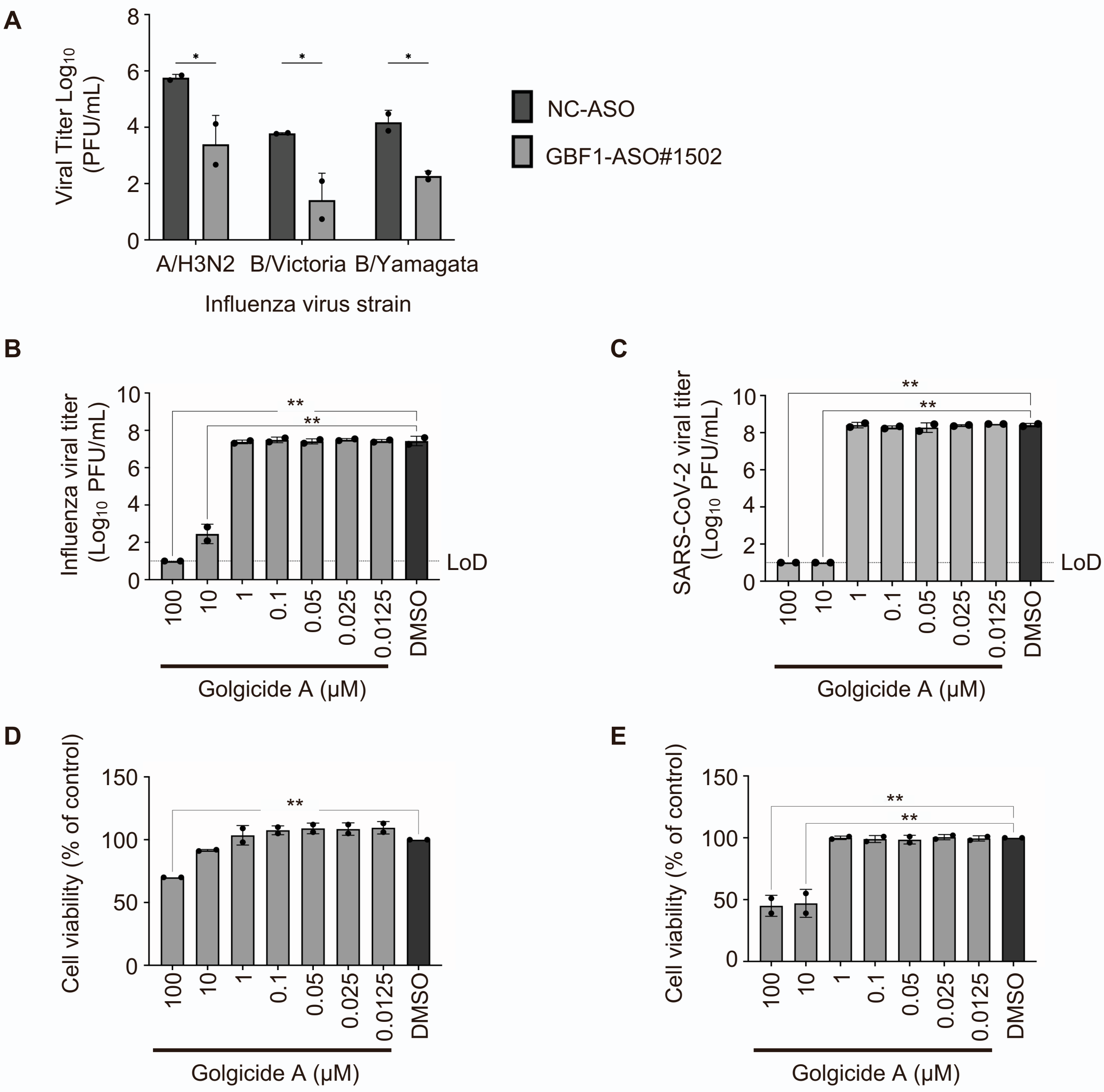

Figure S6. GBF1 has broad-spectrum antiviral activity. Related to Figure 6.
(A) HEK293 cells were reverse-transfected with GBF1-ASO#1502 at a concentration of 100 nM. The following day, the cells were infected with A/Victoria/361/2011(A/H3N2) (50 PFU/ well), B/Tokyo/UT-BB078/2017 (B/Victoria), or B/Massachusetts/02/2012 (B/Yamagata) (1000 PFU/well). Supernatants were collected at 2 dpi and subjected to plaque assay. Each data point represents the mean of duplicate wells from a single independent experiment. Data are presented as the mean ± standard deviation (S.D.) from two independent experiments. Statistical analysis was performed by using a two-way ANOVA followed by Holm-Šídák multiple comparisons test (*p < 0.05). (B—E) WSN virus (B) or SARS-CoV-2 (C) at an MOI of 0.001 was used to infect HEK293 cells (D) or HEK293 A/T cells (E). After a 1-h infection, the cells were washed and incubated with medium containing the indicated concentration of GCA. DMSO (final concentration 0.1%, corresponding to the DMSO concentration in the highest GCA treatment) was used as a control. Culture supernatants were harvested at 48 hpi for influenza and 72 hpi for SARS-CoV-2. Viral titers were measured by use of plaque assays and cell viability was measured by CellTiter-Glo. Data are presented as the mean ± S.D. of triplicate wells from two independent experiments. Statistical analysis was performed using a one-way ANOVA followed by Dunnet's multiple comparisons test (ns: not significant; *p < 0.05; **p < 0.01).

Table S1. Antisense oligonucleotides (ASOs) used in this study

Name	Sequence (5′→3′)
GBF1-ASO#238	CAAACCTGTCAGCGGATC
GBF1-ASO#317	CGGGCATTTCGTTTGATG
GBF1-ASO#321	CCATCGGGCATTTCGTTT
GBF1-ASO#473	GTATCTTCAGAGCGAATC
GBF1-ASO#618	ATCCGTGCCCACAAAACG
GBF1-ASO#812	TGCACCATGTCTACGAGA
GBF1-ASO#874	TGTTGGTCCCCACATAGT
GBF1-ASO#1381	GATTGACGTAATCCATGT
GBF1-ASO#1386	CCGGGGATTGACGTAATC
GBF1-ASO#1502	CGGTCGTGTGGATTGGTG
GBF1-ASO#1505	TGGCGGTCGTGTGGATTG
GBF1-ASO#1508	TTATGGCGGTCGTGTGGA
GBF1-ASO#1516	CCTCTGAGTTATGGCGGT
GBF1-ASO#1632	CTGGAATAAGTGACGGCA
GBF1-ASO#1651	GTCGCTCTATGCTGAGTA
GBF1-ASO#1663	CATAAAGGTTTAGTCGCT
GBF1-ASO#1683	GCATACTCGCAGGGAAGC
GBF1-ASO#1720	ACTTGAGGTGCTCTCGCA
GBF1-ASO#2323	TTAGTTCCCGTGGATCTG
GBF1-ASO#2326	CAATTAGTTCCCGTGGAT
GBF1-ASO#2328	TTCAATTAGTTCCCGTGG
GBF1-ASO#2330	ATTTCAATTAGTTCCCGT
GBF1-ASO#2518	TGCGGTCACTCACAAACT
GBF1-ASO#2522	TTTTTGCGGTCACTCACA
GBF1-ASO#2529	GTCAATGTTTTTGCGGTC
GBF1-ASO#2530	GGTCAATGTTTTTGCGGT
GBF1-ASO#2774	TTACGAACATTGTGGTTG
GBF1-ASO#3148	CATAGTGGGCGGAGATCA
GBF1-ASO#3527	CCCCGAACACTAGACTGC
GBF1-ASO#3784	GGTCTCGAACAGTCTGCC
GBF1-ASO#3793	GGTATAGATGGTCTCGAA
GBF1-ASO#3795	GTGGTATAGATGGTCTCG
GBF1-ASO#3980	AGCCCATACGCAACCTGG
GBF1-ASO#4196	GAAGTGTACCCTCGATCC
GBF1-ASO#4198	CGGAAGTGTACCCTCGAT
GBF1-ASO#4200	GTCGGAAGTGTACCCTCG
GBF1-ASO#4214	GTGTAGACCTCTGAGTCG
GBF1-ASO#4248	TGATCGGTGTATCTTGCC
GBF1-ASO#4314	GGAGTTATCAACGTCATC
GBF1-ASO#5362	AGTCAATGCGTTCCCAGG
GBF1-ASO#5406	GACGGTCTGCTTGAAGAG
GBF1-ASO#5832	TGACCTGCCTTAGTTGAC
NC-ASO#1-AmNA/LNA[18]	**GCT**TGCTCAACTCTA**CG**T
NC-ASO#12-AmNA/LNA[18]	**GGT**TGATCATTGCTG**TC**G

All internucleotide phosphodiester bonds were substituted with phosphorothioate linkages. Underline: 2′,4′-BNA/LNA; C: LNA-5-methylcytidine; bold underline: AmNA; **C**: AmNA-5methylcytidine

Data S1. Raw, uncropped images of the western blotting membranes.

Figure S1A

GBF1

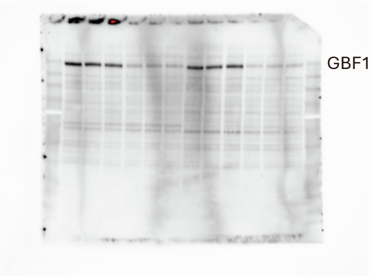

GBF1 marker

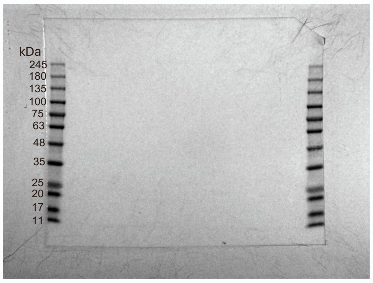

β-Actin

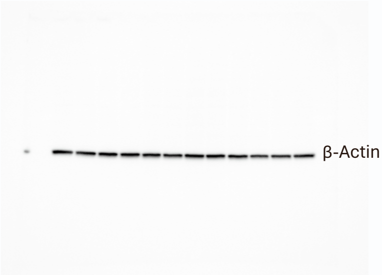

β-Actin marker

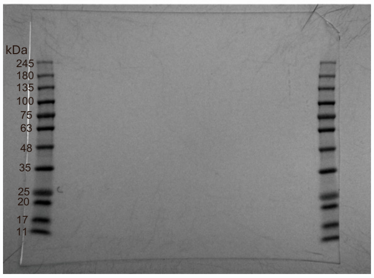

Figure S1B

GBF1

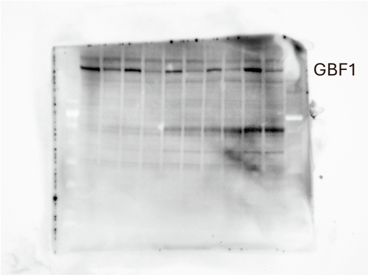

GBF1 marker

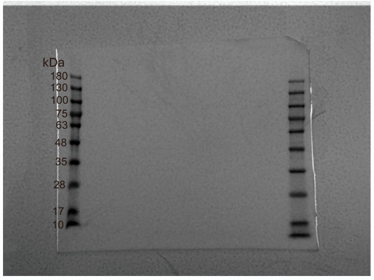

GBF1

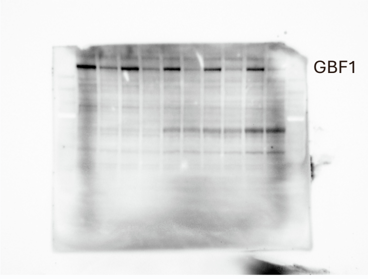

GBF1 marker

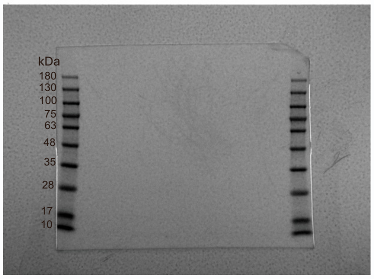

β-Actin

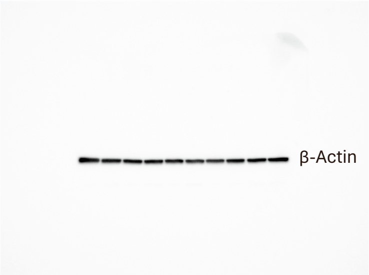

β-Actin marker

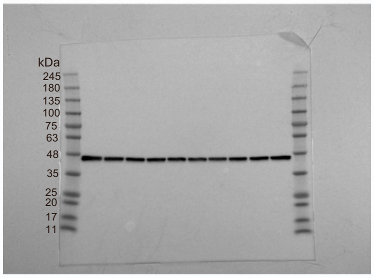

*merge image

Figure S2.

Spike

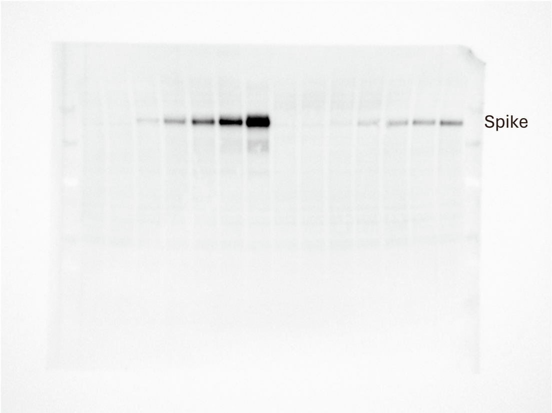

Spike marker

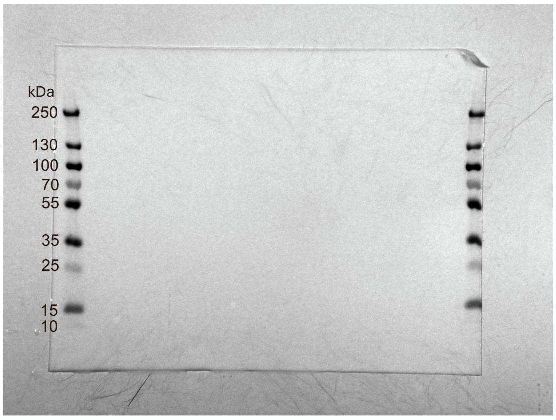

Nucleocapsid

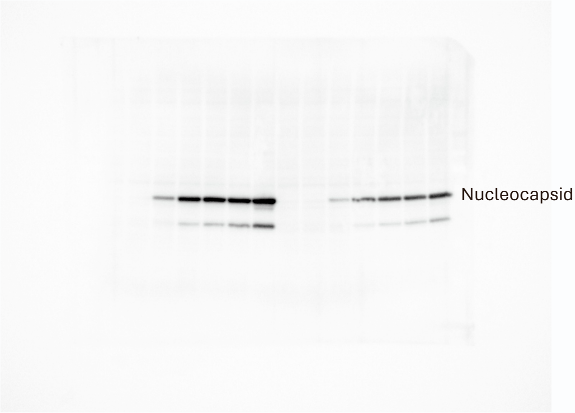

Nucleocapsid marker

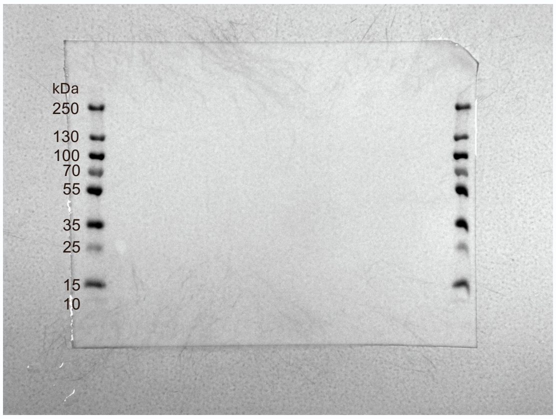

Membrane

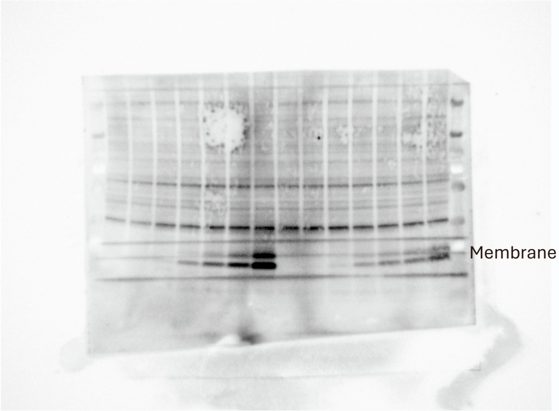

Membrane marker

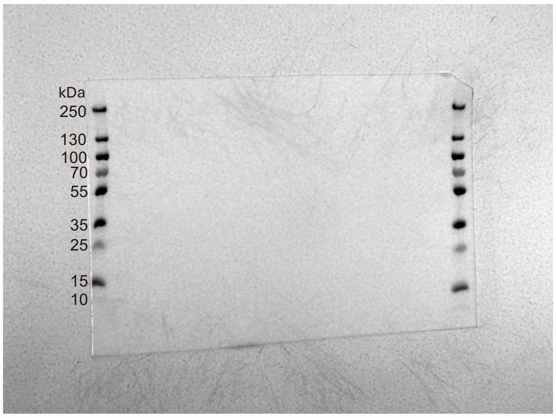

Figure S2.

Envelope

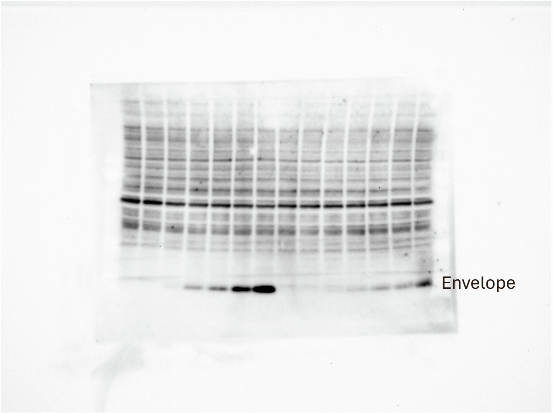

Envelope marker

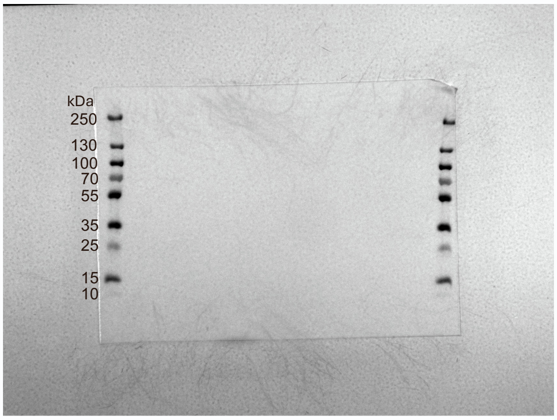

GBF1

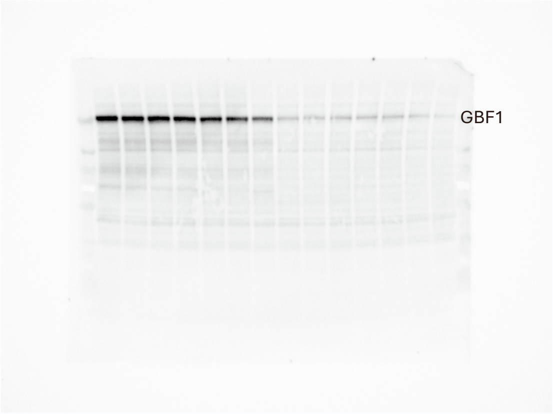

GBF1 marker

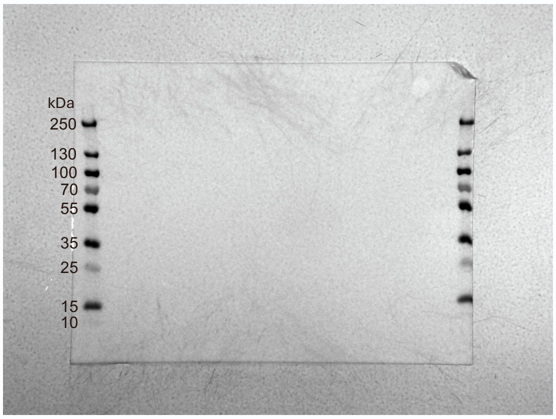

β-Actin

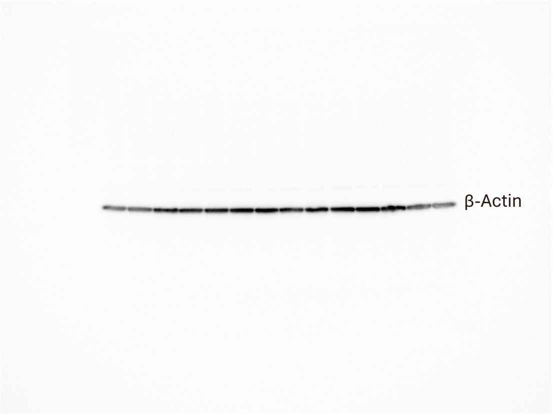

β-Actin marker

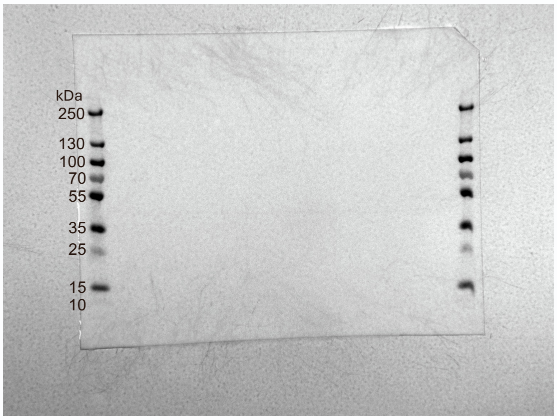

Figure S5A

GBF1

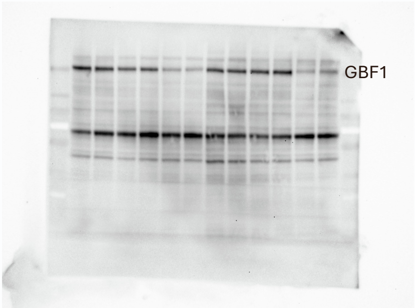

GBF1 marker

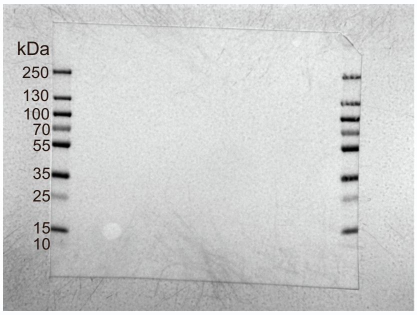

β-Actin

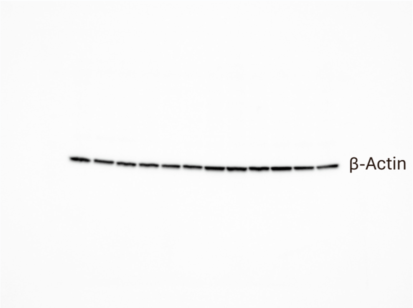

β-Actin marker

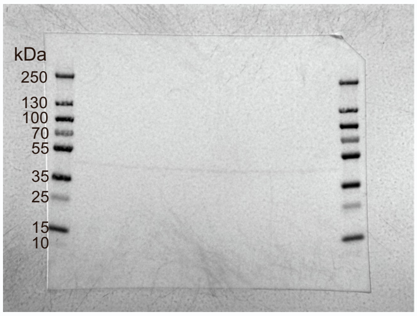

Figure S5B

GBF1

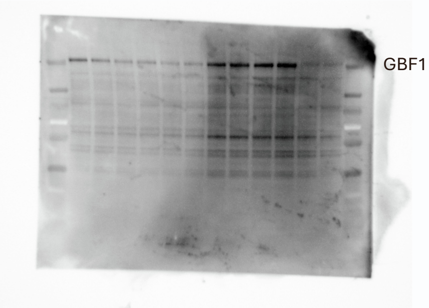

GBF1 marker

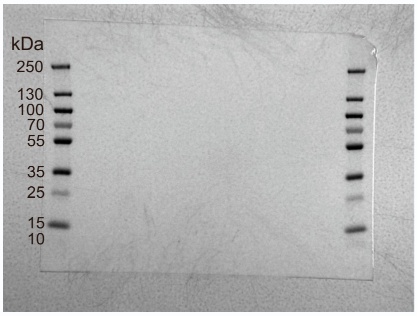

β-Actin

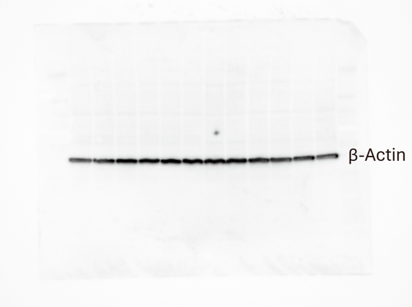

β-Actin marker

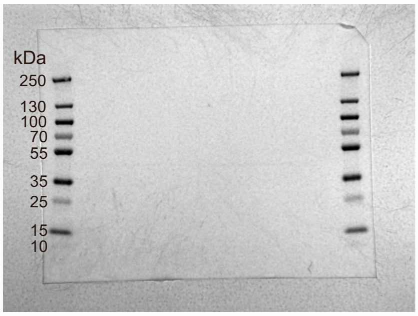

Supplement: Document S1. Figures S1–S6 and Table S1 and Data S1 [file mmc1.pdf]
